# A56 EVALUATING THE DYNAMICS OF *CYP3A11* AND *REG3G* MODULATION BY IL-22

**DOI:** 10.1093/jcag/gwad061.056

**Published:** 2024-02-14

**Authors:** A Delanne-Cuménal, K Flannigan, S Hirota

**Affiliations:** University of Calgary Calvin Phoebe and Joan Snyder Institute for Chronic Diseases, Calgary, AB, Canada; University of Calgary Calvin Phoebe and Joan Snyder Institute for Chronic Diseases, Calgary, AB, Canada; University of Calgary Calvin Phoebe and Joan Snyder Institute for Chronic Diseases, Calgary, AB, Canada

## Abstract

**Background:**

IL-22 is a cytokine produced by a number of immune cell populations, including type 3 innate lymphoid cells. We have shown previously that IL-22 suppresses the expression *Cyp3a11* in the mouse intestine, a gene that encodes a drug metabolizing enzyme that accounts for roughly 70% of total drug metabolism. In addition, IL-22 induces the expression of *Reg3g*, the gene that encodes an antimicrobial C-type lectin that has bactericidal activity against Gram+ bacteria in the small intestine. Modulation of these proteins can influence both the oral bioavailability of drugs and microbial composition.

**Aims:**

The aim of this study was to define the existence of reversible modulation of IL-22 on *Cyp3a11* and *Reg3g* expression in the intestinal epithelium.

**Methods:**

*In vitro* enteroids from each part of the mouse small intestine were cultured in 3D in the presence or absence of IL-22 for 5 days. *Cyp3a11* and *Reg3g* transcript expression was evaluated by qPCR followed by protein analysis to define their modulation at varying timepoints after IL-22 treatment cessation.

**Results:**

IL-22 reduced *Cyp3a11* expression, an effect that normalized the day after treatment cessation. However, 4 days after treatment cessation there was rebound hyperexpression of *Cyp3a11* in duodenal enteroids, which normalized the following day. IL-22 reduced the expression and activity of *Cyp3a11* which normalized soon after stopping IL-22 treatment. In contrast, IL-22 induced the expression of *Reg3g*, an effect that lasted up to 5 days after treatment cessation. The effect of IL-22 on *Cyp3a11* expression and activity was rapidly reversed in contrast to the effect on *Reg3g* which was more prolonged.

**Conclusions:**

IL-22 suppresses the expression of *Cyp3a11* and induces the expression of *Reg3g*. The reversible nature of these effects is different between both, with the increased expression of *Reg3g* 5 days after IL-22 treatment cessation. These data suggest that the regulation of gene expression, protein or enzyme activity by IL-22 may exhibit varying temporal effects and should be considered when assessing its role in intestinal mucosal biology.

**Funding Agencies:**

This work was funded by the Dr. Lloyd Sutherland Chair in IBD/GI Research and the Weston Foundation.

